# Total Sleep Deprivation Impairs Lateralization of Spatial Working Memory in Young Men

**DOI:** 10.3389/fnins.2020.562035

**Published:** 2020-10-06

**Authors:** Ziyi Peng, Cimin Dai, Xiaoping Cai, Lingjing Zeng, Jialu Li, Songyue Xie, Haiteng Wang, Tianyi Yang, Yongcong Shao, Yi Wang

**Affiliations:** ^1^School of Psychology, Beijing Sport University, Beijing, China; ^2^Department of Cadra Word 3 Division, PLA Army General Hospital, Beijing, China; ^3^Suzhou Institute of Biomedical Engineering and Technology, Chinese Academy of Sciences, Suzhou, China; ^4^China Institute of Sports and Health Science, Beijing Sport University, Beijing, China; ^5^State Key Lab of Space Medicine Fundamentals and Application, China Astronaut Research and Training Centre, Beijing, China

**Keywords:** sleep deprivation, spatial working memory, event-related potentials, lateralization, n-back

## Abstract

Total sleep deprivation (TSD) negatively affects cognitive function. Previous research has focused on individual variation in cognitive function following TSD, but we know less about how TSD influences the lateralization of spatial working memory. This study used event-related-potential techniques to explore asymmetry in spatial-working-memory impairment. Fourteen healthy male participants performed a two-back task with electroencephalogram (EEG) recordings conducted at baseline and after 36 h of TSD. We selected 12 EEG points corresponding to left and right sides of the brain and then observed changes in N2 and P3 components related to spatial working memory. Before TSD, P3 amplitude differed significantly between the left and right sides of the brain. This difference disappeared after TSD. Compared with baseline, P3 amplitude decreased for a duration as extended as the prolonged latency of N2 components. After 36 h of TSD, P3 amplitude decreased more in the right hemisphere than the left. We therefore conclude that TSD negatively affected spatial working memory, possibly through removing the right hemisphere advantage.

## Introduction

Sleep quality and time spent sleeping have decreased gradually with increasing social pressure and acceleration of life pace in recent years; however, sleep loss negatively affects productivity and sleep disturbance can lead to various diseases. Total sleep deprivation (TSD) damages brain function, leading to deficits in alertness, attention, and working memory (Durmer and Dinges, 2005; Lim and Dinges, 2010; Basner et al., 2013). Post-TSD decreases in short-term memory are associated with abnormal functional connections between the hippocampus and the cerebral cortex (Li et al., 2016). After TSD, participant responses to working memory tasks were significantly slower (Gujar et al., 2010). Furthermore, positive activation of the medial parietal cortex was significantly reduced, negative activation of the anterior medial frontal lobe and posterior cingulate decreased, while activation of left dorsal lateral prefrontal and bilateral thalamus increased (Gujar et al., 2010). Total sleep deprivation also causes declines in learning and memory (Kusztor et al., 2019).

Working memory is a system that temporarily stores and maintains a limited amount of information to complete a specific task. Working memory consists of the central executive, visuospatial sketchpad, phonological loop, and episodic buffer (Baddeley, 1992). The visuospatial sketchpad comprises two independent parts: visual working memory and spatial working memory. Spatial working memory involves retaining the locations where objects have appeared (Huntley et al., 2011). A topic of interest in cognitive neuroscience is therefore the activation of specific brain regions during these various working-memory processes.

Functional asymmetry is a very important feature of human brain function, with one cerebral hemisphere controlling particular tasks more than the other. This asymmetry is particularly evident in language, visual spatial processing, and emotional expression. Thus, clarifying the nature of this asymmetry is critical for understanding functional neural-processing organization (Ma and Han, 2012). Available neuropsychological research indicates that the right hemisphere of the brain has the advantage in attention to spatial positioning. The right hemisphere is effective in coding, emotional expression, processing and synthesis of overall visual spatial stimulation and configuration information (Thoma et al., 2014). This gives the right hemisphere an advantage in processing non-verbal, synthetic, and intuitive information. Damage to the right hemisphere tends to cause syndromes with lateral negligence and loss of vision on one side (Mesulam, 1999; Danckert and Ferber, 2006). Additionally, the posterior dorsal parietal lobe participates in storage of spatial working memory, while the frontal lobe played is involved in information processing (Pierard et al., 2011). Finally, activation intensity of the dorsolateral prefrontal cortex increases with increasing working memory load (Chee et al., 2006; Lythe et al., 2012). Taken together, we speculate that spatial tasks in working memory mainly activate the right hemisphere.

Many event-related-potential (ERP) studies have used N2 and P3 waves as a potential physiological indicator to reflect TSD effects. Reduced amplitude and sustained latency of the P3 and N2 component are associated with prolonged sobriety (Koslowsky and Babkoff, 1992; Morris et al., 1993; Jones and Harrison, 2001). A decrease in P3 volatility may reflect a reduction in individual attention and discernment of target stimuli (Zhang et al., 2019). Research in the field of sleep using these techniques has linked TSD with response inhibition and working memory, and shown that working memory would be impaired after TSD. In addition, most of these studies have a pre-post-design (Kjellberg et al., 1977; Venkatraman et al., 2011). However, we know less about whether TSD has an effect on the dominant functional areas of certain cognitive processes, specifically whether a lack of sleep would alter functional asymmetry in spatial working memory. There is a functional compensation mechanism in the brain (Drummond et al., 2004). During language learning tasks, a dynamic compensatory change between the prefrontal cortex and parietal area may occur after sleep deprivation (Drummond et al., 2000). Whether impaired spatial working memory after sleep deprivation is characterized by asymmetry or because of the brain’s functional compensation to maintain the original characteristics also needs further discussion.

In this study, we designed a two-back spatial working memory task and used a pre-post-design to explore whether TSD impairs the right-hemisphere advantage of spatial working memory. To examine variation in functional asymmetry after TSD, we recorded electroencephalogram (EEG) data to evaluate changes in N2 and P3 waves. It was hypothesized that there is a right hemisphere advantage in spatial working memory. After sleep deprivation, the spatial working memory capacity is overall impaired, and the right hemisphere advantage is also affected.

## Materials and Methods

### Participants

Sixteen healthy right-handed male adults (age range: 21–28 years, mean age: 23 years) participated in the study. All possessed good sleep habits (PSQI < 5) and no mental or physical illness. Additionally, they had normal vision or corrected vision >1.0, as well as intelligence scores > 110 on the Raven test. An experimenter explained all procedures before the experiment. For 2 weeks, subjects slept regularly for 7–9 h per day. From 48 h prior to the start of the experiment, participants did not smoke, drink alcohol or coffee and did not consume any medication. Written informed consent was obtained from all participants. The experiment was approved by the Ethics Committee of Fourth Military Medical University.

### Experimental Task

**FIGURE 1:**
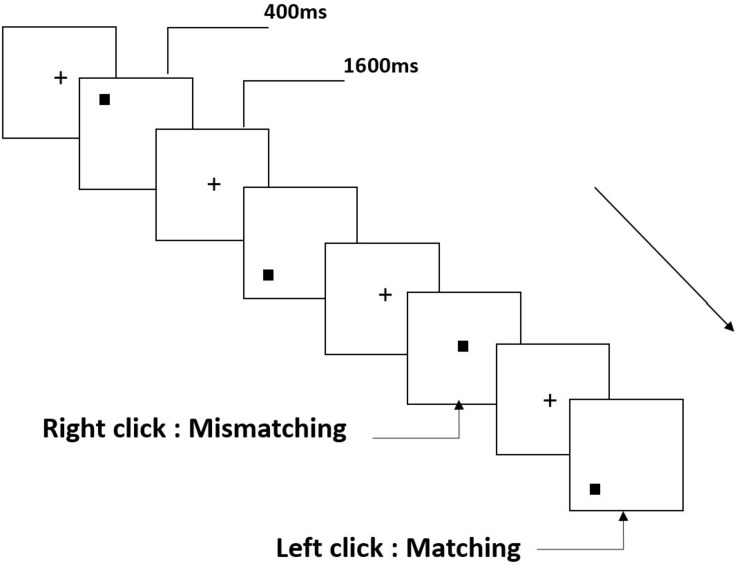
Schematic diagram of spatial working memory task.

All participants were instructed to complete a two-back task for spatial working memory (Figure 1). Stimuli are small black squares, shown on a white background (3 × 3 grid) with an approximate visual angle of 0.4 × 0.4° (width: 0.5 cm, height: 0.5 cm). The task involved 122 trials, each presenting the target for 400 ms; the stimulus onset asynchronous (SOA) time with white “+” was 1600 ms. Participants were asked to left-click the computer mouse if the current target matched the stimulus presented two trials earlier, and right-click if the stimuli did not match. “Matching” and “mismatching” conditions were presented pseudorandomly in a 1:1 ratio.

### Experimental Procedures

**FIGURE 2:**
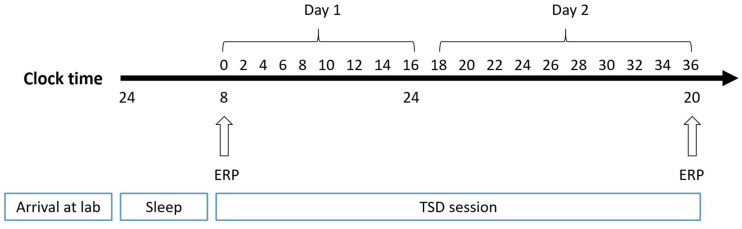
Experimental design. After 8 h of sleep in lab, participants experienced 36-h TSD. The arrows indicate the time points during the 2-back spatial working memory task and EEG recording simultaneously.

The experiment adopted a pre-post design, which is widely used in sleep research. The participants made two visits to the laboratory. In the first visit, they were instructed to practice spatial working memory tasks until they achieved 90% accuracy. Then, they visited the laboratory for a second time the day before the experiment and slept there. At 8:00 AM the next day, subjects performed the spatial working memory task, while experimenters simultaneously recorded the baseline EEG. The second EEG recording was performed after 36 h of TSD in the laboratory (Figure 2). During sleep deprivation, central inhibitory or stimulant drugs were prohibited. Subjects were paired and undertook experiments simultaneously. The nursing staff accompanied participants throughout the TSD session for observation and to prevent them from falling asleep.

### EEG Recordings

Continuous scalp EEG was recorded using electrode caps placed at 32 locations of the 10–20 system, along with SynAmps2 amplifiers. The reference is mean mastoids (A1 and A2), and grounded electrodes were placed on the forehead. Recordings occurred at 1000 Hz and all channel impedance was kept below 5 K-Ω. Four additional electrodes were placed above and below the left eye to record bipolar vertical and horizontal electrooculograms.

### Data Analysis of Behavioral Experiments

Data from two subjects were excluded due to technical errors, leaving *n* = 14 for statistical analyses using repeated-measures ANOVA, with Greenhouse-Geisser non-sphericity correction and Bonferroni *post hoc* test. Data are represented as means and standard deviation (SD).

### EEG Data Analysis

Pre-analysis of EEG data was conducted in Scan 4.3. Eye-moment artifacts were corrected with ocular artifact reduction. Epochs with a length of 900 ms (range: −100 ms to 800 ms) at stimulus onset were extracted from EEG data. For each trial, the system automatically rejected voltage exceeding ±100 μV. Epoch data were filtered with a bandpass filter from 0.05 to 30 Hz, and frequency slope was 24 dZB/oct. The components related to the correct response were calculated for further analysis.

Components related to the correct response were calculated. The P3 component was measured as maximum positive values from time windows of 250–450 ms. The N2 component was measured as minimum negative values from time windows of 150–350 ms. Only channels F7, F8, F3, F4, FT7, FT8, FC3, FC4, T7, T8, C3, and C4 were statistically compared here. All ERP results were analyzed using repeated-measures ANOVA to determine the main effects and interactions between deprivation states (baseline and 36-h TSD), hemispheres (left: F7, F3, FT7, FC3, T7, C3 and right: F8, F4, FT8, FC4, T8, C4), and channels (F7, F8; F3, F4; FT7, FT8; FC3, FC4; T7, T8; C3, C4). This also included Greenhouse-Geisser corrections for non-sphericity and Bonferroni *post hoc* tests and simple effects.

## Results

### Behavioral Performance

The results of the behavioral experiments are presented in Table 1. The mean reaction time was longer at 36-h TSD than at baseline (ANOVA, *F*_[1,13]_ = 1.038, *P* = 0.327), but the difference was not significant. After 36-h TSD, the accuracy rate of spatial working memory was significantly decreased (*F*_[1,13]_ = 10.465, *P* = 0.007). During 36-h TSD, the main effect of time significantly influenced the correct number per unit time (*F*_[1,13]_ = 5.111, *P* = 0.042).

**TABLE 1:** Behavioral data (mean ± SD) on the 2-back task at baseline and after 36-h TSD.

	Baseline	36-h TSD
Mean reaction time(ms)	497.85 ± 82.70	520.50 ± 90.91
Correct rate(%)	0.95 ± 0.04	0.86 ± 0.11
Correct number/sec	1.96 ± 0.33	1.72 ± 0.40

### Amplitude

The means and standard deviations of the amplitudes at the F7, F8, F3, F4, FT7, FT8, FC3, FC4, T7, T8, C3, and C4 electrodes during TSD are listed in Table 2.

**TABLE 2:** The average peak amplitude of the N2 and P3 components in the correct response condition across multiple electrode sites at baseline and after 36-h TSD.

		Baseline		36-h TSD	
		N2	P3	N2	P3
F7	M ± SD	−3.27 ± 2.55	5.44 ± 2.47	−2.42 ± 2.69	2.82 ± 3.44
F8	M ± SD	−3.23 ± 3.75	6.11 ± 3.69	−2.84 ± 3.38	3.63 ± 3.52
F3	M ± SD	−3.92 ± 4.45	7.85 ± 3.77	−2.63 ± 4.10	4.75 ± 4.85
F4	M ± SD	−3.06 ± 5.05	9.46 ± 4.59	−2.74 ± 4.70	5.76 ± 5.24
FT7	M ± SD	−3.08 ± 2.07	6.30 ± 2.68	−2.02 ± 2.47	3.28 ± 4.06
FT8	M ± SD	−2.91 ± 3.06	6.16 ± 3.44	−2.65 ± 3.26	3.33 ± 3.53
FC3	M ± SD	−3.79 ± 5.99	8.58 ± 4.06	−2.17 ± 4.21	6.21 ± 5.05
FC4	M ± SD	−1.94 ± 5.96	10.74 ± 5.15	−1.68 ± 5.46	7.02 ± 5.88
T7	M ± SD	−2.81 ± 2.22	6.94 ± 2.37	−1.90 ± 2.42	4.14 ± 3.55
T8	M ± SD	−1.97 ± 2.65	7.35 ± 3.18	−1.54 ± 2.56	4.51 ± 3.33
C3	M ± SD	−2.64 ± 6.47	9.87 ± 4.14	−1.84 ± 4.33	7.09 ± 4.42
C4	M ± SD	−1.02 ± 5.25	11.36 ± 4.47	−0.88 ± 4.33	8.20 ± 4.85

**FIGURE 3:**
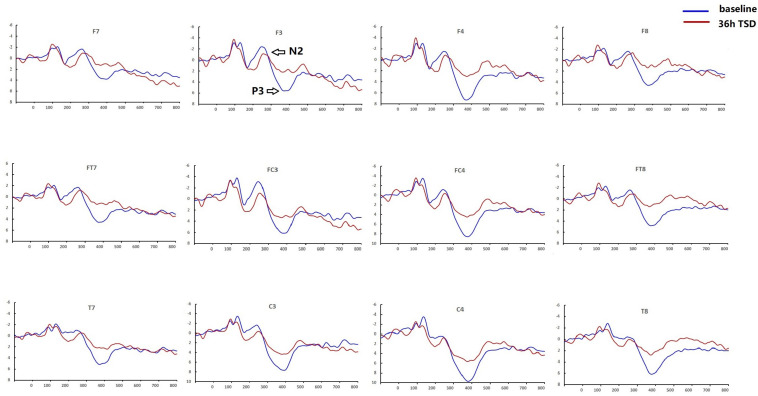
Comparison of ERP amplitude and latency between baseline and 36-h TSD for the correct response in working memory. The channels are ordered left-right and top-bottom as follows: F7, F3, F4, F8, FT7, FC3, FC4, FT8, T7, C3, C4, T8.

**FIGURE 4:**
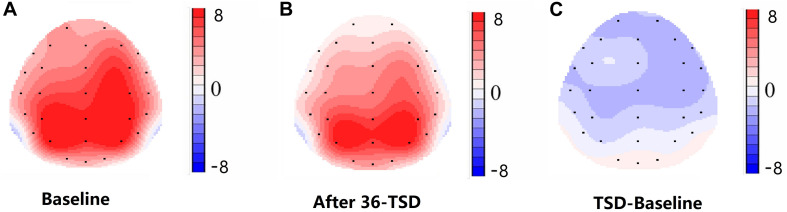
Topographic map of correct response during working memory task across different sleep conditions **(A–C)**. **(A)** P3, 250–450 ms, at baseline. **(B)** P3, 250–450 ms, after 36-h TSD. **(C)** P3, 250–450 ms, 36-h TSD baseline subtracted.

Compared with baseline, P3 amplitude decreased significantly after 36-h TSD (*F*_[1,13]_ = 26.880, *P* = 0.000), but while N2 amplitude decreased after 36-h TSD, the difference was not significant (*F*_[1,13]_ = 1.064, *P* = 0.321) (Table 2; Figure 3). Significant main effects of hemispheres on the P3 amplitude (*F*_[1,13]_ = 6.077, *P* = 0.028) were found during the TSD condition (Figure 4).

For the P3 amplitude, the interaction between deprivation states (baseline vs. 36-h TSD) and hemispheres (left vs. right) was not significant (*F*_[1,13]_ = 0.694, *P* = 0.420). However, simple effect analysis revealed that before sleep deprivation, P3 amplitude was significantly greater in the right hemisphere than in the left (*P* = 0.016). After sleep deprivation, this asymmetry disappeared (*P* = 0.128). In addition, we found that the amplitude of P3 was observed most significantly in C3 and C4 (*F*_[__5,65__]_ = 33.347, *P* = 0.000) (Figure 3). No other main effects or interaction effects reached statistical significance.

### Latency

The means and standard deviations of the latencies at the F7, F8, F3, F4, FT7, FT8, FC3, FC4, T7, T8, C3, and C4 electrodes during TSD are listed in Table 3.

**TABLE 3:** The average peak latency of the N2 and P3 components in the correct response condition across multiple electrode sites at baseline and after 36-h TSD.

		Baseline		36-h TSD	
		N2	P3	N2	P3
F7	M ± SD	258.78 ± 32.42	386.19 ± 19.63	262.25 ± 33.26	357.57 ± 36.11
F8	M ± SD	248.88 ± 30.21	381.27 ± 23.64	256.38 ± 23.43	376.36 ± 46.88
F3	M ± SD	248.81 ± 25.17	377.42 ± 25.61	270.75 ± 18.97	364.46 ± 36.61
F4	M ± SD	240.10 ± 26.12	372.92 ± 24.58	264.88 ± 24.77	376.11 ± 34.38
FT7	M ± SD	244.88 ± 27.67	380.31 ± 26.50	270.21 ± 28.03	369.86 ± 40.02
FT8	M ± SD	249.37 ± 37.18	381.46 ± 21.96	249.00 ± 33.81	360.82 ± 42.98
FC3	M ± SD	252.51 ± 17.91	372.19 ± 26.02	261.17 ± 20.83	374.50 ± 29.33
FC4	M ± SD	243.42 ± 20.35	373.46 ± 28.62	266.29 ± 19.65	367.54 ± 36.23
T7	M ± SD	244.64 ± 21.59	379.23 ± 17.48	262.79 ± 33.48	375.18 ± 29.81
T8	M ± SD	242.46 ± 36.71	387.50 ± 17.35	251.88 ± 35.74	362.82 ± 41.29
C3	M ± SD	244.85 ± 27.23	375.23 ± 27.17	246.21 ± 26.78	369.36 ± 28.83
C4	M ± SD	232.26 ± 23.02	371.08 ± 28.84	231.75 ± 36.54	358.50 ± 39.21

The latency of N2 (*F*_[1,13]_ = 9.106, *P* = 0.010) was significantly prolonged after TSD (Table 3; Figure 3). Although P3 latency was prolonged after 36 h-TSD, the difference was not significant (*F*_[1,13]_ = 2.734, *P* = 0.122). In addition, we found the shortest latency of N2 in C3 and C4 (*F*_[__5,65__]_ = 4.144, *P* = 0.002) (Figure 3). No other main effects or interaction effects reached statistical significance.

The N2 and P3 amplitudes and latencies that were elicited at the 12 electrode sites are presented in Figure 3. The topographic map of the correct response in the working memory task in different sleep conditions (baseline, 36-h TSD and the difference between the two conditions) is presented in Figure 4.

## Discussion

In this study, we successfully used ERP analysis to determine the effects of TSD on spatial working memory. Changes in performance during memory tasks were consistent with impaired spatial working memory: participants experienced a decline in accuracy. Prolonged sleep deprivation lowers awakening levels and elevates the awakening threshold, thus hampering work performance (Sanders, 1983; Corsicabrera et al., 1999). Even if an individual remains alert, response time is significantly longer when fatigued than when not fatigued. This phenomenon is related to reduced efficiency in reaction selection and execution after sleep deprivation (Schacht et al., 2008). In this study, the mean reaction time tended to be longer at 36-h TSD than at baseline, but the difference was not significant. When sleep is insufficient, people tend to conservatively estimate their performance. This tendency may increase the likelihood of participating in compensatory behaviors, which may protect against the negative consequences of TSD (Boardman et al., 2018). Furthermore, in terms of ERP indicators, we also observed lower amplitude and greater latency in N2 and P3 waves related to spatial working memory.

The N2 component reflects selective attention and processing of emotional stimuli (Zhang et al., 2014). Here, we demonstrated a significant prolongation of N2 latency, but no significant change in amplitude. Previous results suggest that prolonged N2 latency reflects increased response time after sleep deprivation (Jin et al., 2015). The brain’s compensatory functions under limited cognitive resources (Gosselin et al., 2005) may explain the absence of a significant change in amplitude.

We also found that P3 amplitude decreased significantly after 36 h of TSD, consistent with previous results. This decrease confirmed that decision-making in cognitive matching is damaged after sleep deprivation (Panjwani et al., 2010). Sleep deprivation can severely impair continuous attention (Huntley et al., 2011) and other cognitive functions that depend on mental or cognitive abilities (Thomas, 2003; Jarraya et al., 2013). The P3 component reflects deployment of attention resources, and its latency indicates the time window for stimulus classification and evaluation. For example, increased P3 latency is associated with impaired discernment of target stimuli (Kutas et al., 1977). Therefore, we believe that 36-h TSD impairs the individual’s spatial working memory ability by affecting the allocation of attention resources, and the individual has a reaction disorder to the change in information (Chua et al., 2014; Honn et al., 2020; Skurvydas et al., 2020). Under baseline conditions, P3 amplitude was significantly greater in the right hemisphere than in the left. This result corroborates the idea that the right hemisphere has a functional advantage when processing spatial information (Li et al., 2016).

Both acute mild sleep loss and extended TSD will undoubtedly cause an increase in sleepiness and decrease in general central nervous system arousal (Schneider and Fisk, 1984; Cote et al., 2009). Studies have shown that after lack of sleep, individuals’ performance in completing the psychomotor vigilance task (PVT) significantly decreases, and both subjective and objective vigilance are negatively affected (Hoedlmoser et al., 2011; Stojanoski et al., 2019). As our results show, after 36 h of sleep deprivation, both behavioral and ERP indicators demonstrated impairment of individual cognitive function. Being awake for a long time changes the activation of the brain’s default network and negatively impacts the balance of functional relationship among different brain networks (Wirsich et al., 2018); poor performance after TSD is related to the separation of task goals and inattention (Drummond et al., 2005). Evidence from fMRI suggests that reduced automatic control ability after TSD may be related to a decrease in the activity and/or functional connection of the cerebellar network (Wang et al., 2014). For working memory tasks, the effects of repetitive partial sleep deprivation (PSD) and TSD are not related to the level of execution load, and the effects of PSD are observed to be small (Lo et al., 2012). Although in this study we only selected spatial working memory tasks, sleep deprivation also reduces the quality of information storage in pronunciation working memory (Zhang et al., 2019) and damages the performance of the entire working memory system (Peng et al., 2020).

The disappearance of a right-hemisphere advantage is the principal finding of our study. However, for the amplitude of P3, the interaction between deprivation states (baseline vs. 36-h TSD) and hemispheres (left vs. right) was not significant. In spite of that, from the results of simple effect analysis, we found a right-hemisphere advantage at baseline. However, after sleep deprivation, this asymmetry disappeared. According to the scalp topography, sleep-deprivation-associated changes to the P3 component were greater in the right hemisphere than in the left hemisphere. This outcome may be attributed to the effect that sleep deprivation has on alertness and spatial attention orientation (Mesulam, 1999; Basner and Dinges, 2005). Alertness is a kind of higher internal arousal state obtained and maintained by the individual. The processing of alertness is divided into phasic alertness and continuous tonic alertness (Raz and Buhle, 2006). Extensive research evidence suggests that alertness is mainly controlled by the right frontal parietal cortex (Fan et al., 2002; Petersen and Posner, 2012). Brain injury studies have shown that impaired right hemispheres damage alertness systems more than impaired left hemispheres (Fernandez-Duque and Posner, 2001). Additionally, the two forms of alertness (phasic and tonic) have significant advantages in the right hemisphere of the brain (Sturm et al., 2006; Fan et al., 2009). Neuroimaging and transcranial direct-current-stimulation studies of normal individuals have also revealed that the right hemisphere is more involved in attention distribution to the left and right visual fields (Corbetta et al., 1993; Chambers et al., 2004). Moreover, the ventrolateral frontal cortex is involved in relocating potential stimuli and has a right hemisphere advantage, in contrast with the frontoparietal network responsible for selective attention (Shulman et al., 2010). Visual spatial attention selectively activates the right parietal cortex of the brain (Corbetta, 2000). Considering these findings in combination with our results, we believe that sleep deprivation impairs spatial attention and alertness system, which may be the internal mechanism of spatial working memory damage after sleep deprivation, and the sleep-deprivation-induced impairment of spatial working memory is asymmetric, occurring mainly in the right hemisphere. Although sleep loss of less than 36 h can affect cognition function (Kusztor et al., 2019; Stojanoski et al., 2019), whether it will significantly impair the superiority of the right hemisphere in processing spatial working memory task still needs to be further explored.

Our study has some limitations. First, we only used a two-back task and did not compare working-memory performance across different difficulties (e.g., three- or four-back). Therefore, we cannot address how workload might influence the negative effects of sleep deprivation. Second, because we only used male volunteers, we hesitate to extend our conclusions to women; future research could increase female subjects to study related issues. There are reasons to believe gender differences may exist; fMRI revealed, for instance, that women’s left hemisphere is more active when performing spatial memory tasks, whereas men’s right hemisphere is more active on the right brain when performing spatial memory tasks, and men use integrated strategies more to help remember spatial locations (Frings et al., 2006). Thus, combining our procedure with fMRI would facilitate a clearer picture of sleep deprivation’s effects on working memory. Third, our low sample size may have affected our ability to generate stable results, as we did not find significant differences for N2 amplitude or P3 latency. In addition, due to the limitation of our sample size, we were unable to find any significant interaction effect between deprivation states and hemispheres directly. Finally, circadian biorhythms affect behavioral performance, and these effects differ across individuals (Montplaisir, 1981; Lavie, 2001). We could not rule out effects of circadian rhythms in this study because we did not measure EEG data at the same time points.

## Conclusion

In conclusion, our research showed that sleep deprivation impaired spatial working memory, specifically damaging lateralization (i.e., right-hemisphere advantage). Therefore, we recommend that future studies consider brain asymmetry when investigating cognitive deficits associated with sleep deprivation. Further clarity on this issue would improve the development of measures to reduce adverse health effects associated with sleep deprivation. Overall, this study provides valuable physiological evidence for understanding mechanisms underlying the effects of sleep deprivation on spatial working memory.

## Data Availability Statement

The raw data supporting the conclusions of this article will be made available by the authors, without undue reservation.

## Ethics Statement

The studies involving human participants were reviewed and approved by The Fourth Military Medical University. The patients/participants provided their written informed consent to participate in this study.

## Author Contributions

YS designed the study. ZP produced the results and wrote the manuscript. CD, XC, LZ, JL, SX, HW, and TY contributed to the data collection and analysis. YS and YW were the guarantors of this study. All authors listed have approved it for publication.

## Conflict of Interest

The authors declare that the research was conducted in the absence of any commercial or financial relationships that could be construed as a potential conflict of interest.
